# Stress in the metastatic journey – the role of cell communication and clustering in breast cancer progression and treatment resistance

**DOI:** 10.1242/dmm.050542

**Published:** 2024-03-20

**Authors:** Eloïse M. Grasset, Sophie Barillé-Nion, Philippe P. Juin

**Affiliations:** ^1^ Université de Nantes, INSERM, CNRS, CRCI2NA, 44000 Nantes, France; ^2^ Équipe Labellisée LIGUE Contre le Cancer CRCI2NA, 44000 Nantes, France; ^3^ Institut de Cancérologie de l'Ouest, 44805 Saint Herblain, France

**Keywords:** Breast cancer, Metastasis, Cluster, Stress

## Abstract

Breast cancer stands as the most prevalent malignancy afflicting women. Despite significant advancements in its diagnosis and treatment, breast cancer metastasis continues to be a leading cause of mortality among women. To metastasize, cancer cells face numerous challenges: breaking away from the primary tumor, surviving in the circulation, establishing in a distant location, evading immune detection and, finally, thriving to initiate a new tumor. Each of these sequential steps requires cancer cells to adapt to a myriad of stressors and develop survival mechanisms. In addition, most patients with breast cancer undergo surgical removal of their primary tumor and have various therapeutic interventions designed to eradicate cancer cells. Despite this plethora of attacks and stresses, certain cancer cells not only manage to persist but also proliferate robustly, giving rise to substantial tumors that frequently culminate in the patient's demise. To enhance patient outcomes, there is an imperative need for a deeper understanding of the molecular and cellular mechanisms that empower cancer cells to not only survive but also expand. Herein, we delve into the intrinsic stresses that cancer cells encounter throughout the metastatic journey and the additional stresses induced by therapeutic interventions. We focus on elucidating the remarkable strategies adopted by cancer cells, such as cell–cell clustering and intricate cell–cell communication mechanisms, to ensure their survival.

## Teaser

**Summary:** This Review examines the multiple challenges cancer cells encounter during metastasis and therapeutic interventions, with a focus on strategies adopted by cancer cells in the context of breast cancer.

## Introduction

Breast cancer stands as the most frequently diagnosed malignancy, retaining its status as a prominent contributor to cancer-related fatalities in women. In-depth molecular assessment of breast tumors has corroborated the established quartet of breast cancer subtypes previously characterized via immunohistochemistry (Box 1): luminal A and B tumors, characterized by the expression of estrogen and progesterone receptors and by differing proliferation rates; *HER2* (*ERBB2*)-amplified tumors; and the triple-negative entity. Although this classification loosely aligns with clinical behavior and treatment responses (Box 1), it falls short in capturing the intricate inter- and intra-tumoral diversity of primary tumors at presentation (reviewed by Nolan et al., 2023). This also extends to their clonal evolution during disease progression or exposure to therapeutic interventions. Analogous to other solid tumors, breast tumors can be envisioned as ecosystems that encompass an intricate interplay of cancer cells arising from the mammary gland and a diverse array of non-cancerous counterparts, all embedded within a remodeled extracellular matrix (ECM).

**Box 1. Breast cancer clinical groups and treatments**

Breast tumors are initially categorized by histology (*in situ* or invasive) and immunochemistry [estrogen receptor (ER or ESR1), progesterone receptor (PR or PGR), HER2 receptor, Ki67 index]. This classifies them into four clinical groups: luminal A (45%, low Ki67, ER^+^/PR^+^/HER2^−^), luminal B (25%, higher Ki67, ER^+^/PR^+/−^/HER2^−^), HER2^+^ (15%, *HER2* gene amplification) and triple-negative (15%, lacking ER, PR and HER2) breast cancers.

Primary tumor removal via surgery has been the common initial treatment for all types of breast cancer. More recently, neoadjuvant approaches (preceding surgery) including chemotherapy and/or radiotherapy are used to reduce tumor size and improve resectability. Moreover, neoadjuvant approaches enable effective monitoring and adaptability.

Endocrine therapy – blocking estrogen – is standard for ER^+^ disease, often combined with CDK4/6 inhibitors for metastatic disease. Aromatase inhibitors reduce the production of estrogen but long-term use can lead to resistance, often due to *ESR1* mutations. Another treatment for ER^+^ disease is the use of phosphoinositide 3-kinase (PI3K) inhibitors that target concurrent PI3K–AKT–mTOR activation (Nolan et al., 2023).

HER2^+^ tumors are treated with the HER2-blocking antibody trastuzumab, improving survival. Resistance to trastuzumab can be tackled with CDK4/6 inhibitors or HER2 small-molecule tyrosine kinase inhibitors, including neratinib and tucatinib.

For aggressive tumors (triple-negative and luminal B), genotoxic drugs such as anthracyclines and taxanes are common. Recent research highlights effective antibody–drug conjugates targeting TROP2 coupled with SN-38 for triple-negative metastatic breast cancer (Bardia et al., 2021). Triple-negative breast cancer treatment evolves, as the cancer itself evolves, due to functioning DNA repair and immune evasion. PARP inhibitors exploit DNA repair deficits, whereas immune checkpoint inhibitors (anti-PD-L1 and anti-PD-1 antibodies) synergize with chemotherapy. For instance, neoadjuvant pembrolizumab (anti-PD-1) and chemotherapy increase complete responses and 36-month event-free survival in patients with triple-negative breast cancer (Topalian et al., 2023).

**Fig. 1.**
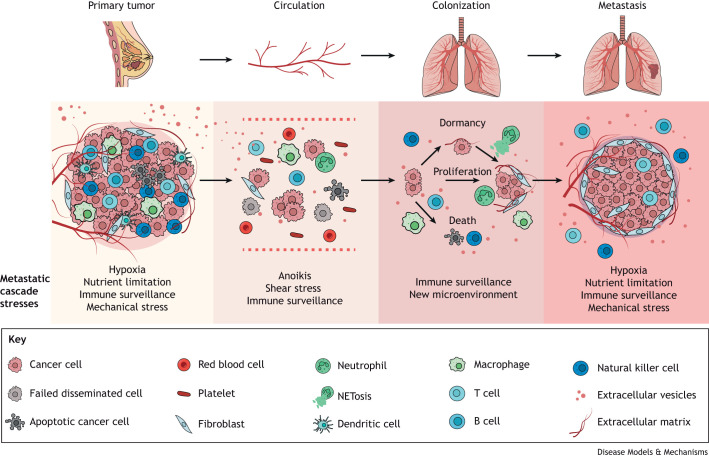
**Cancer cell stresses during the metastatic cascade.** Cancer cells are exposed to numerous stresses they need to counteract to survive and form distant metastasis. The main physiological stresses inherent to cancer development are indicated for each of the distinct steps of the metastatic cascade. Therapy-induced stresses also have effects throughout these steps and include chemotherapies, surgery, radiotherapies and immunotherapies. NETosis, release of neutrophil extracellular traps (NETs).

The apex of the threat of breast cancer lies in its propensity for dissemination, culminating in the ominous formation of metastases. For malignant cells to colonize distant sites, they must first escape from the confines of the primary tumor and infiltrate the bloodstream or lymphatic system. Yet, once ensnared within circulation, cancer cells are subjected to an array of challenges that must be surmounted for their survival and subsequent establishment at remote sites (Fig. 1).

Throughout its progression, a tumor faces an array of physiological stresses, cultivating intrinsic resilience while adapting to its adversarial surroundings. Conventional anticancer agents primarily target rapidly dividing cancer cells, often sparing dormant cells and even the elusive cancer stem cell population(s) (Baldominos et al., 2022; Correia et al., 2021). Paradoxically, in instances of incomplete tumor eradication, the very therapeutic interventions designed to combat the malignancy inadvertently foster tumor evolution via selection of more aggressive subclones and chronic cellular adaptation. An embodiment of this intrinsic adaptation is the epithelial–mesenchymal transition (EMT) cellular program, which can herald the acquisition of an aggressive, therapy-resistant phenotype (Fischer et al., 2015; Goldman et al., 2015).

Our aim here is to dissect the strategies adopted by breast cancers to manage the diverse stresses encountered throughout their journey, from localized primary tumors to the emergence of circulating tumor cells (CTCs) and eventual colonization as metastases of different sizes. Of particular interest is the profound impact of therapeutic pressures, a decisive arbiter of patients' prognoses.

## Physiological stresses in tumorigenesis – implications for primary and secondary tumors

Breast carcinoma cells navigate multiple physiological hurdles en route to becoming a tumor. Beyond inherent genetic anomalies, tumor initiation intricately disrupts the mammary gland equilibrium and manipulates its environment to fuel tumor progression. Notably, cancer cells transform fibroblasts into cancer-associated fibroblasts (CAFs), which are major players in the tumor microenvironment (Albrengues et al., 2015, 2014). Their remarkable plasticity allows them to contribute to ECM remodeling during tumorigenesis, driving fibrosis and cancer cell invasion (Gaggioli et al., 2007; Goetz et al., 2011; Grasset et al., 2018). Moreover, CAFs modulate inflammatory responses and immune reactions by releasing mediators that attract immunosuppressive cells (Costa et al., 2018). CAFs also directly interact with breast cancer cells, creating a favorable niche for malignancy (Bertero et al., 2019; Su et al., 2018) and therapeutic resistance (Louault et al., 2019). Importantly, persistent unresolved inflammation is a common occurrence during tumorigenesis, hampering immune responses by disrupting adaptive innate immune cell communication and facilitating immune evasion (reviewed by Greten and Grivennikov, 2019). Furthermore, the hypoxic conditions often found within solid tumor cores significantly impact both the intrinsic evolution of the tumor and its broader ecosystem, influencing processes such as angiogenesis and metabolism (reviewed by Zhang et al., 2021b). This intricate web of interactions between cancer and non-cancer cells, involving ECM, secreted factors and direct contact, ultimately paves the way for tumor emergence and progression.

Cancer cells, when confronted with the myriad challenges of tumor development and the spread of metastases, often exhibit remarkable plasticity. This refers to their capacity to undergo reprogramming towards different cellular states in response to both internal and external influences (Yuan et al., 2019). One of the primary mechanisms underlying this plasticity in cancer cells is EMT, a phenomenon wherein epithelial cells lose their characteristic features such as cell–cell junctions and cellular polarity, while adopting a mesenchymal phenotype. This transformation is pivotal as it enables cancer cells to downregulate the expression of genes associated with epithelial traits and acquire attributes typically linked to mesenchymal cells, which equips them with the ability to invade surrounding tissues, access the bloodstream and establish secondary tumors at distant locations (Nieto et al., 2016). Conversely, the reverse process, the mesenchymal–epithelial transition (MET), is believed to be advantageous for the growth of metastases (Del Pozo Martin et al., 2015; Esposito et al., 2019; Giampieri et al., 2009; Ocaña et al., 2012; Tsai et al., 2012).

In breast cancer, a nuanced form of EMT is observed. Here, cancer cells exhibit a partial EMT program wherein they maintain the expression of epithelial genes while concurrently acquiring mesenchymal characteristics. This particular phenomenon is crucial for the formation of metastases in triple-negative breast cancer (Grasset et al., 2022). In luminal tumors, a transient EMT process has been demonstrated during the formation of metastases in the genetically engineered mouse model MMTV-PyMT (Li et al., 2020; Pastushenko et al., 2018). In conclusion, for survival amid the myriad stresses during primary tumor formation, cancer cells manipulate their microenvironment and demonstrate remarkable adaptability, particularly through the activation of EMT programs. However, upon departure from the primary site, they face distinct challenges.

## Stresses in circulation

For patients, the most dangerous aspect of cancer is its dissemination and the formation of metastasis. Metastasis to sentinel lymph nodes carries significant implications, including heightened tumor aggressiveness, bleaker prognoses and substantial impact on treatment decisions for patients. To spread in a distant site, cancer cells must leave the primary tumor and intravasate into blood or lymph circulation. Studies using the 4T1 mammary carcinoma cells, a model of triple-negative breast cancer, *in vivo* have provided evidence that cancer cells can infiltrate the bloodstream within lymph nodes (Brown et al., 2018; Pereira et al., 2018). However, phylogenetic analysis of matched primary tumors and metastases has shown that certain breast cancers can metastasize without initially involving lymph nodes (Ullah et al., 2018). Further investigations are imperative to elucidate disparities between how lymph and blood circulation affect CTC survival.

**Fig. 2.**
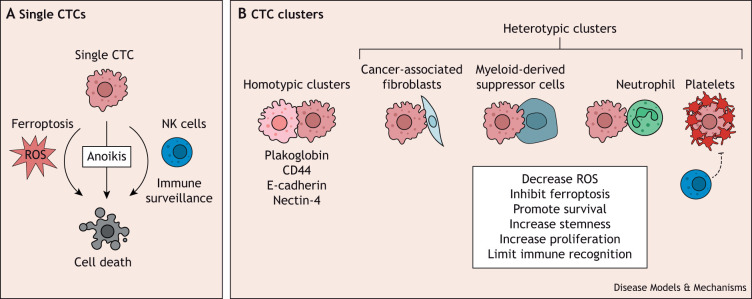
**Stress in the circulation – united we stand, divided we fall.** (A) Single circulating tumor cells (CTCs) often die by anoikis or ferroptosis, or are eliminated via immune surveillance. NK cells, natural killer cells; ROS, reactive oxygen species. (B) However, CTCs can form homotypic (cancer cells only) or heterotypic (cancer and non-cancer cells) clusters to enhance their survival in circulation. Homotypic CTC clusters preserve cell–cell contacts through a variety of complexes, including plakoglobin, homophilic CD44 interaction, E-cadherin and nectin-4. The distinct types of heterotypic CTC clusters observed in patient are also shown. CTC clusters survive in circulation by different mechanisms, including a decrease in internal ROS, inhibition of ferroptosis, increase in the expression of ‘stemness’ genes, increase in the expression of genes implicated in proliferation and a decrease in NK cell recognition.

Once in circulation, cancer cells are challenged by multiple stresses that they need to overcome to survive and colonize a distant site. CTCs face their first significant challenge in the form of anoikis (Fig. 2A), a form of programmed cell death (see Box 2) triggered by the detachment of cells from the ECM or neighboring cells. This protective mechanism typically prevents the survival of detached cells under normal physiological conditions. Anoikis operates through two distinct pathways: one involves mitochondrial disruption via the activation of BH3-only proteins of the BCL2 family, and the other entails the activation of cell surface death receptors following ECM detachment. Both pathways ultimately lead to the activation of caspases and subsequent cell death by apoptosis (Paoli et al., 2013). Although apoptosis is the most rapid mechanism for eliminating cells without proper ECM attachment, it has been revealed that non-apoptotic cell death pathways can step in to impede cell survival when apoptosis is inhibited in ECM-deprived cells (Debnath et al., 2002). For instance, in normal mammary cells, detachment from the ECM triggers alterations in cell metabolism, primarily through the disruption of glucose transport, which subsequently leads to the production of reactive oxygen species (ROS) resulting in cell death (Schafer et al., 2009). Similarly, using patient-derived xenografts, Piskounova et al. (2015) demonstrated that in melanomas, CTCs are exposed to an oxidative stress impairing CTC survival and metastasis formation.

**Box 2. Cell death pathways actionable by anticancer treatments**

Cell death occurs through various mechanisms, each directed by distinct molecular pathways. In contrast to uncontrolled cell death, such as necrosis, regulated cell death follows specific genetic and molecular instructions (Galluzzi et al., 2018). The primary modes of regulated cell death include:

1. **Anoikis:** a form of apoptotic cell death triggered by disruptions in cell–cell or cell–ECM attachments (Paoli et al., 2013). Cancer cells develop resistance to anoikis by altering their integrin repertoire, activating various pro-survival signals and being influenced by the tumor microenvironment, which modulates factors such as ECM stiffness, triggers the epithelial–mesenchymal transition and promotes self-renewal capabilities.
2. **Apoptosis:** a programmed cell death process executed through a cascade of caspase activation, most notably caspase-3 (CASP3), that is triggered via extrinsic or intrinsic (mitochondrial) apoptotic pathways. The extrinsic pathway originates from caspase-8 (CASP8) activation following engagement of cell death receptors. The intrinsic pathway involves mitochondrial outer membrane permeabilization and is regulated by the BCL2 family of proteins (Juin et al., 2013).
3. **Ferroptosis:** a distinct mode of cell death first described by Dixon et al. (2012). It relies on iron-dependent phospholipid peroxidation and is governed by multiple cellular metabolic pathways, including redox homeostasis, iron management, mitochondrial function, and the metabolism of amino acids, lipids and sugars (reviewed by Jiang et al., 2021).
4. **Necroptosis:** a lytic form of cell death dependent on RIPK1 and RIPK3 kinases and the pore-forming protein MLKL. It occurs when apoptotic caspases are blocked or suppressed (Vandenabeele et al., 2023).
5. **Pyroptosis:** another lytic cell death process resulting in the loss of plasma membrane integrity, initially described upon activation of the caspase-1 (CASP1)-dependent inflammasome pathway. It relies on pore-forming gasdermins, which release pro-inflammatory cytokines such as IL-1β and IL-18 (Bertheloot et al., 2021).
  Apoptosis, necroptosis and pyroptosis are frequently interconnected, as common molecular mediators can either bolster or impede one another (Kopeina and Zhivotovsky, 2022). Moreover, each of these modes can activate the integrated stress response or immunogenic cell death.
6. **Integrated stress response:** this relies on a central signaling network that senses cellular stress and operates by reprogramming protein translation. Its regulatory switch centers on the formation of a ternary complex involving the eukaryotic translation initiation factor EIF2 (Costa-Mattioli and Walter, 2020).
7. **Immunogenic cell death:** a form of cell death that activates an immune response. The immunogenicity of a particular type of cell death is determined by the release of antigens and adjuvants during the process (Kroemer et al., 2022, 2013).

Cancer cells, however, have evolved various strategies to evade anoikis. These encompass the overexpression of oncogenes (Gupta et al., 2019; Schafer et al., 2009), the manipulation of pro-survival signals (Zhu et al., 2011), the induction of EMT (Huang et al., 2013), alterations in metabolic pathways (Piskounova et al., 2015; Tasdogan et al., 2020), the formation of membrane blebs (Weems et al., 2023) and the facilitation of cell–cell interactions. Of particular interest is the role of cell clusters in cancer dissemination. CTC clusters can either be composed of cancer cells only (homotypic clusters) or can also include non-cancer cell types (heterotypic clusters).

Multiple studies demonstrated that homotypic cancer cell clusters, defined as groups of two or more cancer cells tightly bound together, exhibit heightened metastatic potential (Thompson, 1974; Watanabe, 1954). These findings are further supported by lineage tracing studies that revealed the multiclonal nature of spontaneous metastases in mouse models. In these experiments, luminal cancer cells from the MMTV-PyMT mouse model (Cheung et al., 2016) or the human triple-negative breast cancer cell line MDA-MB-231 (Aceto et al., 2014) were labelled with different fluorophores and injected into the mammary glands to create multicolor primary tumors. Remarkably, the resulting CTC clusters and metastases also exhibited a multicolored composition, thus confirming the multiclonal nature of metastasis. Deep-sequencing studies have further demonstrated polyclonal metastases in the majority of patients with breast cancer across various subtypes (Hoadley et al., 2016; Siegel et al., 2018; Ullah et al., 2018).

CTC clusters can form when clusters of cancer cells detach from the primary tumor (Aceto et al., 2014; Cheung et al., 2016) or when individual cancer cells detach and subsequently aggregate within the bloodstream (Liu et al., 2019). Although CTC clusters remain a rare occurrence in clinical settings, their presence has been associated with unfavorable patient outcomes, including a decrease in progression-free survival and overall survival (Aceto et al., 2014; Larsson et al., 2018; Mu et al., 2015; Wang et al., 2017a).

### Protection in homotypic CTC clusters

In breast cancers, homotypic CTC clusters demonstrate remarkable resilience against anoikis by preserving cell–cell contacts through a variety of complexes, including plakoglobin (JUP) (Aceto et al., 2014), homophilic CD44 interactions (Liu et al., 2019), E-cadherin (CDH1) (Padmanaban et al., 2019) and nectin-4 (Brown et al., 2018) (Fig. 2B). Intriguingly, distinct survival pathways emerge depending on the specific adhesion complex engaged. In the case of intercellular homophilic CD44–CD44 interactions, these fuel FAK signaling within cancer cell clusters, promoting survival (Liu et al., 2019). Meanwhile, the expression of E-cadherin and the overall cluster configuration counteract TGF-β-induced ROS production, thereby promoting cancer cell survival, enhancing seeding and facilitating metastasis formation. Conversely, removal of E-cadherin exposes detached cells to ROS-induced cell death (Padmanaban et al., 2019). Similarly, Labuschagne et al. (2019) demonstrated that loss of attachment causes mitochondrial perturbations and ROS production in 393T epithelial cells and A549 and HeLa cancer cells. Conversely, cluster conformation induces hypoxia and mitophagy that clear damaged mitochondria and reduce ROS production, therefore promoting cell survival and metastatic capacity. Lastly, Brown et al. (2018) have reported that nectin-4 breast cancer clusters exhibit vulnerability to ferroptosis upon detachment from the ECM. Ferroptosis is a form of regulated cell death characterized by lipid peroxide buildup and iron-related oxidative stress (see Box 2). However, the presence of integrin α6β4 within these clusters activates Src signaling, protecting cancer cells against ferroptosis-induced cell death (Brown et al., 2018).

Intriguingly, a recent study in melanoma has unveiled that exposure to the lymphatic environment protects cancer cells from ferroptosis induced in the bloodstream, thereby promoting their survival during subsequent exposure to the bloodstream (Ubellacker et al., 2020). This research posits the intriguing possibility of distinct survival mechanisms between the blood and lymphatic systems, with the latter remaining relatively underexplored. Further investigations are warranted to unravel the molecular intricacies of CTC survival within the lymphatic milieu and its clinical significance, particularly in breast cancers.

### Protection in heterotypic CTC clusters

Cancer cells evolve in a complex ecosystem, even within the bloodstream. Beyond interactions among cancer cells themselves, CTC clusters engage in complex dialogues with various stromal cells, immune cells and blood components to promote their survival and metastatic colonization (Fig. 2B). A recent study by Szczerba et al. (2019) revealed that approximately 3.4% of CTCs formed heterotypic clusters with white blood cells, predominantly neutrophils. Intriguingly, the presence of CTC–neutrophil clusters in patients with advanced breast cancer correlated with a shorter time to disease progression, in stark contrast with that in patients whose CTCs remained unaccompanied by neutrophil interactions. The study also underscored that CTC–neutrophil clusters exhibited higher proliferative rates and established a greater number of metastatic sites in mouse models compared to solitary CTC clusters (Szczerba et al., 2019). Similarly, CTC clusters with myeloid-derived suppressor cells have been identified in patients with breast cancer and have been shown to actively promote CTC survival, proliferation and successful metastatic colonization (Sprouse et al., 2019).

Furthermore, tumor cells have been observed to engage in interactions with platelets while circulating in the bloodstream. Notably, 30% of patients with solid tumors exhibit thrombocytosis, characterized by an elevated platelet count, which has been correlated with unfavorable clinical outcomes (Stravodimou and Voutsadakis, 2013). Platelets play pivotal roles in promoting metastasis through diverse mechanisms. For instance, Haemmerle et al. (2017) demonstrated that platelets induce resistance to anoikis in ovarian and colon human cancer cell lines by activating YAP1. Additionally, platelets harbor an array of growth factors and cytokines capable of modulating signaling pathways in cancer cells. Remarkably, platelet-derived TGF-β induces EMT in murine colon and breast cancer cells, priming them for metastatic progression (Labelle et al., 2011). Platelets also form a shield around CTCs, protecting them from natural killer (NK) cells (Nieswandt et al., 1999; Palumbo et al., 2005), thereby aiding CTCs in evading immune surveillance. NK cells are innate lymphoid cells with the ability to rapidly eliminate malignant cells. Recently, Lo et al. (2020) showed that NK cells preferentially target monoclonal metastases arising from single CTCs as opposed to polyclonal metastases originating from CTC clusters in mouse models of breast cancers. This preference was attributed to elevated expression of cell–cell adhesion and epithelial genes within CTC clusters, coupled with decreased expression of NK cell-activating ligands. Inducing MET in CTCs reduced NK ligand expression, subsequently decreasing their sensitivity to NK-mediated cytotoxicity.

Interestingly, researchers have also observed the formation of heterotypic clusters in which CTCs join forces with CAFs. The prevailing hypothesis posits that CAFs, dislodged from primary tumors, hitch a ride with CTCs, traveling to metastatic sites and fortifying cancer cell survival and the establishment of metastases (Ao et al., 2015; Duda et al., 2010; Hurtado et al., 2023; Sharma et al., 2021). These findings collectively suggest that targeting the clustering of CTCs with other cells could potentially mitigate metastasis.

### Escape from shear stress

In circulation, various factors such as flow rates, vessel dimensions and shear stress exert critical influences on the survival of cancer cells. Shear stress, determined by the frictional forces exerted by fluid on cancer cells, presents distinct magnitudes within different types of vessels, ranging from relatively high in arterial flow (4-30 Dyn cm^−2^) to lower in venous (1-4 Dyn cm^−2^) and very low in lymphatic flow (0.64-12 Dyn cm^−2^) (Follain et al., 2020). Elevated shear stress, characteristic of arterial flow, can provoke cell cycle arrest (Chang et al., 2008) and inflict physical damage leading to apoptosis and necrosis (Lien et al., 2013). Regmi et al. (2017) proposed that an increase in bloodstream shear stresses similar to levels experienced during intensive physical exercise might induce CTC necrosis. However, these results were obtained from an *in vitro* microfluidic modeling system and need to be validated *in vivo*, as the duration of cancer cell exposure to shear stress appears to be a pivotal determinant of cell death, and numerous studies have established a short half-life for CTCs, spanning from 40 s to 30 min (Aceto et al., 2014; Hamza et al., 2021). To endure shear stress, CTCs can deploy a mechano-adaptive response through activation of the RhoA–actomyosin signaling axis (Moose et al., 2020). Furthermore, it has been demonstrated that CTC association with platelets can shield them from the detrimental effects of shear stress (Egan et al., 2014).

CTC clusters from the triple-negative breast cancer cell line MDA-MB-231 have a faster clearance rate from the bloodstream than that of single CTCs in mice (Aceto et al., 2014). A potential explanation for the shorter circulation time of CTC clusters involves their physical entrapment in small vessels and capillaries. Given their larger size, it is believed that clusters encounter greater difficulty passing through capillaries than that encountered by individual CTCs (Phillips et al., 2015). However, studies using microfluidic systems have shown that patient-derived CTC clusters can reconfigure into single-file chains within capillary-sized vessels, and a similar reconfiguration was observed in zebrafish models (Au et al., 2016). In addition, Allen et al. (2019) reported no significant difference in extravasation rates between single CTCs and CTC clusters generated from HeLa cells. Live-imaging investigations in zebrafish have revealed that CTC clusters from breast and melanoma cancers can extravasate through a process known as angiopellosis. This entails the dynamic remodeling of the vascular wall around CTCs and their active expulsion from the vascular lumen into the surrounding tissue (Allen et al., 2019; Follain et al., 2018). Following successful arrest within the vasculature – mediated either by occlusion or active adhesion to the vessel wall – CTCs are exposed to intensified shear forces. Intravital imaging studies in mouse lungs have unveiled that these elevated shear forces can fragment arrested CTCs, generating tumor microparticles that recruit myeloid cells, thereby promoting metastatic colonization (Entenberg et al., 2018; Headley et al., 2016).

## Stress associated with metastatic colonization

**Fig. 3.**
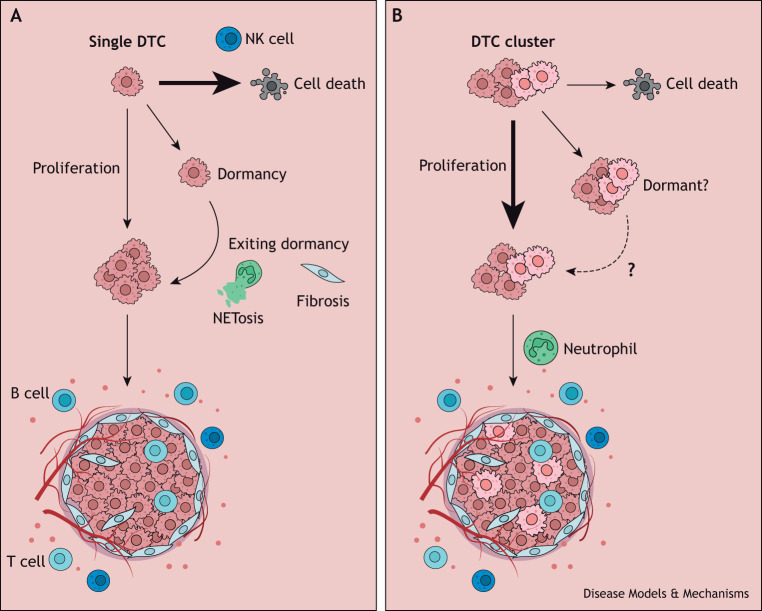
**Disseminated cancer cells and metastasis formation.** (A) Most single disseminated tumor cells (DTCs) die within hours after extravasating into a new tissue due to an inhospitable environment and immune clearance. DTCs that survive this first step of colonization can either proliferate or enter dormancy, notably to avoid immune recognition. Multiple factors secreted by the host organ or cancer cells themselves promote dormancy and survival of dormant DTCs. Although the mechanisms implicated in the awakening of dormant DTCs are not fully understood, it has been shown that inflammation promotes dormant DTC proliferation due to neutrophil activation and the production of neutrophil extracellular traps (NETs), a process known as NETosis. Other mechanisms implicated are fibrosis and aging, notably with the production of the Wnt ligand sFRP1 by aged fibroblasts. (B) Unlike single DTCs, DTC clusters have been shown to colonize distant sites more efficiently and to resist apoptosis upon extravasation, notably due to their ability to form nanolumens in which the cancer cells secrete epigen, and due to the initiation of epigenetic reprogramming promoting the expression of stemness and proliferation genes. Bone marrow-derived mesenchymal stromal cells can also induce pro-tumorigenic and pro-metastatic neutrophils that promote macrometastatic outgrowth. Although it is not clear yet whether DTCs clusters can enter dormancy, the multiclonality of these clusters can offer a survival and proliferation advantage.

Following their successful detachment from the primary tumor and survival through the numerous challenges encountered in the circulation, cancer cells face a daunting task: colonizing an unfamiliar microenvironment. This final step represents the most stressful and inefficient phase of the metastatic cascade. Several studies have illustrated that, upon injection into the bloodstream, the majority of cancer cells manage to extravasate and endure within a new organ for a brief period. However, only a small fraction can persist over an extended duration and eventually proliferate to establish metastatic growths (Cameron et al., 2000; Koop et al., 1995; Luzzi et al., 1998; Varghese et al., 2002). During this ultimate stage of the metastatic process, cancer cells must surmount a multitude of challenges, including infiltrating foreign tissues, eluding the immune defenses, occasionally adopting a latent tumor-initiating state, and ultimately thriving to form significant macrometastases (Fig. 3). The timelines for these steps can extend over months to years, contingent upon the specific cancer subtype. In breast cancer, for instance, the triple-negative subtype often exhibits relapse within 5 years after surgery, whereas hormone receptor-positive breast cancer may manifest relapses after a decade (Copson et al., 2013; Kennecke et al., 2010; van Maaren et al., 2019). Although it has not been conclusively established, a plausible rationale for the observed variations among breast cancer subtypes lies in their distinct capacity to adapt to stresses. Contrary to luminal and HER2-enriched breast cancer subtypes, triple-negative breast cancer does not rely on hormone signaling to sustain proliferation, which gives it an advantage to propagate in multiple environments. In addition, triple-negative tumors are enriched for hybrid epithelial–mesenchymal cells, a phenotype associated with plasticity (Grasset et al., 2022). Finally, another possible explanation could be that patients with hormone receptor-positive breast cancer benefit from extended periods of 5 to 10 years of endocrine therapy and patients with HER2-positive breast cancer receive anti-HER2-targeted therapies, whereas patients with triple-negative breast cancer rely predominantly on acute interventions such as surgery, chemotherapy and radiation owing to the absence of specific receptors (see Box 1). For instance, the introduction of the anti-HER2-targeted therapy trastuzumab has significantly enhanced survival rates of patients with HER2-positive breast cancer (Romond et al., 2005), which was previously comparable to that of patients with triple-negative breast cancer.

### Colonization of a foreign tissue

Once they extravasate, CTCs are referred to as disseminating tumor cells (DTCs). Most DTCs die of apoptosis within hours after arrival to their metastatic site due to the unfavorable environment (Wong et al., 2001). This may be hostile to DTCs due to various factors, including an inappropriate ECM, deficiencies in nutrients and chemokines, the anatomical and histological defensive mechanisms of the host organ, and immune defenses. For instance, in the brain, astrocytes have been shown to promote DTC death by releasing the pro-apoptotic cytokine FasL (FASLG) (Valiente et al., 2014).

Several studies have revealed that, in addition to promoting survival in circulation, clusters of cancer cells play a pivotal role in promoting metastatic colonization and expansion. Using the tail-vein injection assay of cancer cell clusters from the MMTV-PyMT mouse model and human breast cancer cell lines, researchers demonstrated that these clusters exhibited notable resistance to apoptosis when seeding the lungs, contrasting with that of solitary cancer cells, which frequently undergo apoptosis (Aceto et al., 2014; Wrenn et al., 2020). A recent study by Wrenn et al. (2020) revealed that clusters of breast tumor cells from the basal-like breast cancer subtype (a molecular subtype of triple-negative breast cancers) create nanolumens, which are sealed reservoirs within the clusters, where cancer cells secrete the growth factor epigen (EPGN) to sustain their proliferation. Additionally, breast cancer cell clustering has been linked to DNA methylation patterns that promote stemness and proliferation, two vital attributes for successful metastasis formation (Gkountela et al., 2019).

Furthermore, the presence of multiple genetic clones within a DTC cluster can significantly contribute to the process of metastatic colonization. For example, in melanoma, *in vivo* experiments using zebrafish models have shown that invasive cells can bind to cells with a more proliferative but less invasive phenotype, forming multiclonal clusters that facilitate the delivery of the proliferative cells to the metastatic site (Campbell et al., 2021). Similarly, in colorectal cancer, more aggressive cancer cells can travel with and enable the colonization of less metastatic cells by priming the metastatic site via fibrosis (Kok et al., 2021). In ovarian cancer, Naffar-Abu Amara et al. (2020) revealed that transient exposure to the growth factor amphiregulin (AREG) produced by non-tumorigenic clones within multiclonal clusters was necessary to permit metastasis formation. In breast cancer, a recent study demonstrated that minor subclones expressing IL11 and FIGF (or VEGFD) indirectly cooperate to promote metastatic progression by modulating bone-marrow-derived mesenchymal stromal cells. These stromal cells induce pro-tumorigenic and pro-metastatic neutrophils that promote macrometastatic outgrowth (Janiszewska et al., 2019).

Surviving DTCs, often referred to as cancer stem cells owing to their resemblance to adult stem cells, sometimes exploit the supportive environments provided by host organ stem cell niches to maintain their stem-like characteristics. These niches orchestrate various signaling pathways, including the Notch, Wnt/β-catenin and BMP pathways, and those of several growth factors such as FGF, IGF and members of the TGF-β family (Mancini et al., 2021; Mannino et al., 2022). For example, breast cancer cells have been observed to metastasize to bone marrow niches, with the expression of CXCR4 in breast cancer cells marking and mediating metastasis to CXCL12-rich bone marrow sites (Müller et al., 2001). In triple-negative breast cancer, CAFs secrete CXCL12 within the primary tumor, promoting the expansion of cancer cell clones that will preferentially metastasize to the CXCL12-rich bone marrow environment (Zhang et al., 2013). The binding of cancer cells to E-selectin (SELE) in the bone vascular niche promotes MET and activates the Wnt pathway to facilitate bone metastasis outgrowth (Esposito et al., 2019). Surprisingly, a study demonstrated that the bone microenvironment can epigenetically reprogram cancer cells that have established there and induce them to travel further, forming tertiary metastases (Zhang et al., 2021a).

In the context of metastasis to the brain, cancer cells from various origins have been shown to establish around brain vasculature, attaching to vessel stem cell niches and basement membranes (Carbonell et al., 2009; Kienast et al., 2010). These cancer cells are capable of modifying the perivascular niche before their arrival, promoting vascular leakiness to facilitate extravasation (Huang et al., 2009) and stimulating fibronectin (FN1) production to establish a pro-metastatic milieu (Murgai et al., 2017). Alongside brain metastases, the process of vessel co-option, whereby metastases invade pre-existing vessels from the host tissue, is a phenomenon frequently observed in lung and liver metastases (Bridgeman et al., 2017; Frentzas et al., 2016; Stessels et al., 2004; Valiente et al., 2014). This process, as demonstrated *ex vivo* using microfluidic devices containing vessels and breast cancer organoids (Silvestri et al., 2020), is thought to be responsible for resistance to anti-angiogenic therapies.

### Metastatic niches

Beyond utilizing the existing adult stem cell niches of the distant organ, DTCs can create their own metastatic niche. Using an elegant labeling strategy, Ombrato et al. (2019) demonstrated that breast cancer DTCs promote a regenerative-like program in lung epithelial cells during their colonization (Ombrato et al., 2019). Upon arriving in the lungs, DTCs also produce the ECM protein TNC, a component of the mammary stem cell niche, to sustain stem cell signaling within cancer cells, thereby promoting DTC survival and facilitating metastatic colonization (Oskarsson et al., 2011). TNC has also been shown to activate perivascular macrophages in the lungs, which subsequently modify endothelial cells to create a vascular niche, similarly to what we discussed above, conducive to metastatic colonization (Hongu et al., 2022). Interestingly, in lung and melanoma cancers, bone marrow-derived hematopoietic progenitor cells expressing VEGFR1 arrive at distant sites before cancer cells to establish a permissive niche for DTCs (Kaplan et al., 2005). Subsequent studies revealed that breast and rhabdomyosarcoma cancer cells from the primary tumor prompt the differentiation of these progenitor cells into myeloid-derived suppressor cells at early metastatic sites, fostering an immunosuppressive pro-metastatic microenvironment within the pre-metastatic niche of distant tissues (Giles et al., 2016). Capitalizing on this recruitment of myeloid-derived suppressor cells into the pre-metastatic niche, Kaczanowska et al. (2021) used genetically engineered myeloid cells (GEMys) to deliver the anti-tumor cytokine IL-12 (IL-12-GEMy). Their promising results demonstrated that IL12-GEMy treatment reverses immune suppression within the metastatic niche, activating antigen presentation and T cell responses, ultimately reducing metastatic and primary tumor burdens and enhancing the survival of rhabdomyosarcoma tumor-bearing mice.

To establish a pre-metastatic niche, cancer cells must communicate with distant sites before the arrival of DTCs. They achieve this by secreting extracellular vesicles and cytokines that modify gene expression in distant sites, promoting ECM remodeling, inflammation, and immune suppression (Hoshino et al., 2015; Peinado et al., 2012; Rodrigues et al., 2019). For example, Gui et al. (2020) recently demonstrated that melanoma tumor-derived factors induce the activation of p38α kinase (MAPK14) in lung fibroblasts, leading to ECM remodeling and the expression of chemokines that attract neutrophils, thereby promoting cancer cell colonization and metastasis.

Although colonization of distal sites remains an inefficient and stress-rich process, the research we discuss here demonstrates how DTCs deploy several strategies to morph the hostile environment to one that is more permissive for their survival, colonization and eventual establishment of clinically relevant (macro)metastases.

### Adaptations to metabolic stress

Contrasted with the primary tumor, each metastatic site presents distinct nutrient compositions and environmental stressors (discussed above). This underscores the imperative for DTCs to adapt metabolically to their new surroundings during colonization and outgrowth. Most proliferating cells, such as cancer cells, rely on the tricarboxylic acid (TCA) cycle to produce the building blocks for growth, a process called anaplerosis. Pyruvate carboxylase (PC) is a key enzyme refilling the TCA cycle by converting pyruvate to oxaloacetate. Glutamine is also important for the TCA cycle. Using *in vivo* ^13^C tracer analysis of 4T1 breast cancer-derived lung metastases compared to that of their primary tumors, Christen et al. (2016) demonstrated that lung DTCs activate pyruvate carboxylase-dependent anaplerosis due to a high abundance of pyruvate in lung interstitial fluid. A follow-up study revealed that breast cancer cells rely on pyruvate to drive collagen-based remodeling of the ECM in the lung metastatic niche (Elia et al., 2019). Contrary to lungs, the brain interstitial environment is highly abundant in both glutamine and branched-chain amino acids (Yudkoff et al., 1993). Brain metastatic cancer cells from triple-negative models (4T1 and MDA-MB-231) were shown to acquire the ability to use branched-chain amino acids and glutamine to produce glucose and proliferate in the brain (Chen et al., 2015). Furthermore, triple-negative breast cancer cells were shown to express N-methyl-D-aspartate receptors and form pseudo-tripartite synapses with glutamatergic neurons in the brain to access glutamate secreted by presynaptic neurons (Zeng et al., 2019). In an elegant study, the authors isolated brain metastatic cells that were either synchronous (rapid recurrence), dormant or metachronous (delayed recurrence) from athymic mice that had been orthotopically implanted with the HER2^+^ HCC1954 and SKBR3 cell lines, and they revealed that metabolic diversity and plasticity within brain-tropic cells determine metastatic fitness (Parida et al., 2022). These studies indicated that cancer cells can adapt their metabolism to the nutrients available at the metastatic site and that their metabolism determines their outgrowth.

### Evading immune defenses

Within the new microenvironment, DTCs become vulnerable to immune surveillance. As we discussed previously, each organ has a distinct microenvironment and innate immune cell composition, which influences the colonization of cancer cells. NK cells play a pivotal role in antimetastatic immune surveillance, with the depletion of NK cells promoting metastasis in various cancer models (Hanna and Burton, 1981; Riccardi et al., 1980; Smyth et al., 1999). Recent findings by Vyas et al. (2023) have highlighted the complementary roles of NK and T cells in melanoma metastasis. NK cells inhibit metastatic colonization while promoting the recruitment and infiltration of CD4^+^ and CD8^+^ T cells into metastatic foci in the lungs. In turn, T cells restrain metastatic outgrowth (Vyas et al., 2023). Further studies are needed to determine whether NK and T cells play similar roles in breast cancers. In breast cancer, however, silencing the IRF7 pathway in tumor cells promotes bone metastasis by evading immune responses mediated by CD8^+^ T and NK cells (Bidwell et al., 2012). In the liver, NK cells express the proapoptotic ligand TRAIL (TNFSF10), inhibiting metastasis formation of TRAIL-sensitive cancer cells, such as the fibrosarcoma cell line L929 and renal adenocarcinoma cell line Renca (Takeda et al., 2001). In addition, liver-resident macrophages, known as Kupffer cells, were shown to limit metastasis of melanoma and colon carcinoma cell lines in the liver through dectin-2 (CLEC6A)-dependent phagocytotic activity (Kimura et al., 2016).

To evade immune clearance, cancer cells employ various strategies. In breast cancers, cancer cells can reprogram tissue-resident immune cells, such as NK cells and macrophages, into a pro-metastatic state (Chan et al., 2020; Sousa et al., 2015). HER2^+^ brain DTCs were showed to secrete lactate, which inhibits NK cell cytotoxicity (Parida et al., 2022). Additionally, cancer cells can shape their microenvironment to create an immune-suppressive milieu. For instance, CAF-derived TGF-β signaling restrains CD8^+^ T cells in the peritumoral stroma, protecting cancer cells from T cell-mediated cytotoxicity by reducing direct cell–cell contact (Mariathasan et al., 2018; Tauriello et al., 2018). CAFs can also foster an immunosuppressive environment by attracting T cells and promoting their differentiation into regulatory T cells expressing FOXP3 (Costa et al., 2018).

Cancer cells themselves can evade the immune system by downregulating their tumor-specific antigens (Anagnostou et al., 2017). Another mechanism involves cancer cells acquiring immune inhibitory receptors at their surface. For example, squamous cell carcinoma tumor-initiating cancer stem cells have been shown to express the surface ligand CD80, which dampens cytotoxic T cell activity when engaging with the immune checkpoint factor CTLA4 (Miao et al., 2019). Induction of EMT in breast and esophageal cancer cells is associated with immune evasion, notably through the expression of another immune checkpoint factor, PD-L1 (or CD274) (Alsuliman et al., 2015; Chen et al., 2017). Intriguingly, the mesenchymal-like state of breast cancer cells closely correlates with the expression of ligands that activate NK cell cytotoxicity (Lo et al., 2020). Another mechanism that allows cancer cells to avoid immune clearance is entering a slow-dividing state. In HER2^+^ breast cancer, DTCs expressing SOX2 and SOX9 can evade NK cell-mediated clearance by entering quiescence and downregulate cell surface-innate immune sensors, a phenomenon known as tumor dormancy (Malladi et al., 2016).

### Exit from tumor dormancy regulated by cellular communications

After successfully colonizing a metastatic site, cancer cells face two possible fates: they can either proliferate and form a tumor mass or they can enter a state of dormancy (Fig. 3). Cancer cell dormancy can result from a delicate equilibrium between proliferation and apoptosis, influenced by factors such as immune defenses (Koebel et al., 2007), the availability of nutrients and growth factors (Holmgren et al., 1995), or signals from the microenvironment (Bragado et al., 2013). This dormancy state is intricately regulated by the microenvironment. For example, endothelial cells in the lung vasculature secrete TSP1 (THBS1), which induces quiescence of breast cancer cell lines (HMT-3522 T4-2 and MDA-MB-231) both *in vitro* and *in vivo* upon injection into mice (Ghajar et al., 2013). Recent research has also revealed that lung epithelial cells induce the production of the Wnt signaling modulator SFRP2 in cancer cells. This stimulates fibronectin production and organization, promoting the survival of the dormant D2.0R breast cancer cell line (Montagner et al., 2020). Furthermore, lung cells secrete BMP that contributes to 4T07 breast cancer cell dormancy (Gao et al., 2012). Type III collagen derived from cancer cells is another key factor in maintaining tumor dormancy of multiple cancer cell lines (including breast cancer) in the lungs. Disruption of collagen III can trigger cancer cell proliferation through DDR1-mediated STAT1 signaling (Di Martino et al., 2022). It remains unclear whether DTC clusters enter a state of dormancy or whether this phenomenon is reserved for single DTCs.

The mechanisms underlying the awakening of dormant cancer cells, their resumption of proliferation and their development into metastases are still not well understood. Recently, it was demonstrated that the STING pathway is a suppressor of metastatic outbreak as it induces immune clearance of metastases. STING (or STING1) activity increases in lung adenocarcinoma cells that re-enter the cell cycle and is dampened by hypermethylation of the *STING* promoter and enhancer in breakthrough metastases (Hu et al., 2023). In the lungs, inflammation can induce NETosis, the release of neutrophil extracellular traps (NETs). These NETs cleave laminin and stimulate the proliferation of dormant breast cancer cells by activating integrin α3β1 signaling (Albrengues et al., 2018). Another factor involved in the reactivation of dormant breast cancer cells is the secretion of the TGF-β antagonist Coco (DAND5) by cancer cells themselves (Gao et al., 2012). In the liver, hepatic stellate cells suppress NK cell-sustained dormancy of breast cancer cells, thus promoting liver metastasis (Correia et al., 2021). Additionally, changes in the microenvironment that occur with aging may explain the reawakening of cancer cells years after treatment. In melanoma, for instance, age-induced reprogramming of lung fibroblasts increased secretion of the soluble Wnt antagonist sFRP1. This inhibited WNT5A in melanoma cells, enabling efficient metastatic outgrowth (Fane et al., 2022). A recent study using orthotopic injections of hormone receptor-positive mouse breast cancer cell lines into syngeneic mice also demonstrated that microenvironmental changes due to aging or fibrotic injury support proliferation and outgrowth of DTCs. Here, the cancer cells upregulate PDGF-C and activate the stroma (Turrell et al., 2023). There is an urgent need to counteract latent DTC awakening to improve patient outcomes. Most treatments, which are discussed in the following section, target highly proliferative cells, often sparing latent DTCs.

## Therapeutic stresses on primary and secondary tumors

### Tumor resection by surgery

Surgical removal of the primary tumor, sometimes accompanied by lymph node removal, typically serves as the frontline approach for patients with breast cancer, aiming to eliminate tumor cells. However, this procedure can inadvertently stimulate metastasis by mechanically disseminating cancer cells or awakening dormant DTCs due to surgery-triggered wound-healing responses, systemic inflammation and immunosuppression (Krall et al., 2018). Using the aggressive D2A1 murine mammary carcinoma cell line expressing GFP, Krall et al. (2018) generated syngeneic tumors of which outgrowth was restricted by a robust GFP-specific T cell response. In this model, surgical wounding by a simple skin incision or subcutaneous implantation of a sponge promoted the outgrowth of distant immunogenic tumors. The authors showed that surgical wounding induced a systemic inflammatory response and that the use of anti-inflammatory treatments prevented surgery-induced tumor growth. We can speculate that this systemic inflammation induced by surgery could suppress the immune control of CTCs and dormant DTCs and therefore promote metastasis. Accordingly, peri-operative anti-inflammatory treatments using anti-inflammatory drugs (Panigrahy et al., 2019) or inhibiting the macrophage receptor ChemR23 (CMKLR1) (Lavy et al., 2023) have shown significant reductions in metastasis formation in multiple animal models, including the 4T1 syngeneic breast cancer model.

### Local radiotherapy

Most patients with cancer undergo radiotherapy, which stands as one of the most effective non-surgical methods for achieving localized tumor control. Over recent decades, remarkable technological progress and enhanced imaging precision have substantially elevated the accuracy, effectiveness and patient comfort associated with radiotherapy. Nonetheless, the potential for radiation-induced damage to non-target tissues still exists, occasionally leading to localized injury. Prior investigations, using preclinical models or clinical data, proposed that suboptimal radiotherapy might foster metastasis. This can occur through the alteration of cancer cells, promoting invasion, migration or adhesion, and by modifying the microenvironment to create a favorable niche, involving endothelial cells and fibroblasts (reviewed by Vilalta et al., 2016). More recently, an analysis of women with breast tumors that were clinically and pathologically similar uncovered a noteworthy trend: a notably higher occurrence of ipsilateral pulmonary metastasis, which occurred on the same side as the breast cancer, in patients who underwent post-operative radiotherapy compared to those who had surgery alone (Nolan et al., 2022). Furthermore, through multiple mouse models of breast cancer (4T07, 4T1 and MMTV-PyMT), this study illustrated that exposure of healthy lung tissue to a single 13 Gy dose of focused radiation or three fractionated doses of 4 Gy specifically to the thoracic cavity triggered a response primarily driven by neutrophils, which locally activate and influence lung epithelial cells, ultimately enhancing the process of metastatic colonization (Nolan et al., 2022). It is crucial to acknowledge that the doses employed in this study exceed those typically received by the lungs in patients with breast cancer. Nonetheless, it is worth considering that even low doses of radiotherapy may have the potential to modify healthy tissue and potentially contribute to metastasis.

### Cytotoxic treatments

Conventional chemotherapies retain significance in breast cancer treatment, especially for aggressive tumors such as triple-negative breast cancer or in metastatic contexts (see Box 1). Although often administered post resection to consolidate cell eradication, their neoadjuvant use is growing, which allows researchers to assess their anti-tumor efficacy. These chemotherapies can activate distinct programmed cell death pathways in cancer cells, including apoptosis, pyroptosis, necroptosis or ferroptosis (see Box 2). Cancer cell death can sometimes lead to the release of damage-associated molecular patterns, promoting immune cell activation, a process called immunogenic cell death. This immunogenic cell death induces anti-tumor immune responses and therefore enhances chemotherapy efficacy (reviewed by Kroemer et al., 2022). For instance, doxorubicin and cyclophosphamide, also used to treat patients with breast cancer, were shown to induce immunogenic cell death in colon carcinoma and thymoma (Casares et al., 2005; Schiavoni et al., 2011). Importantly, programmed cell death pathways interplay and can converge to oxidative and endoplasmic reticulum stress, along with DNA damage responses, finally culminating in the integrated stress response, which initially aids cell survival and eventually eliminates cells that cannot adapt (Costa-Mattioli and Walter, 2020; Wang et al., 2017b). Mitochondria, pivotal in cellular life and regulated cell death, orchestrate programmed cell death. Their actions, driven by BCL2 family proteins that regulate mitochondrial outer membrane permeabilization (MOMP), also activate proinflammatory signaling, such as the cGAS/STING pathway via mitochondrial DNA release (Rongvaux et al., 2014; White et al., 2014), causing either inflammatory or immunologically silent cell death (Marchi et al., 2023; Li et al., 2023). However, chronic cGAS/STING activation was shown to promote breast cancer metastasis in mice implanted with the MDA-MB-231 and 4T1 breast cancer cell lines (Bakhoum et al., 2018). Strikingly, a paracrine effect mediated by FGF2 during mitochondria-dependent apoptosis in HeLa cells may foster survival of neighboring cells (Bock et al., 2021). Thus, depending on the cellular context or death pathways triggered by anticancer agents, pro- or anti-tumoral effects may arise and modulate tumor response to anticancer treatments.

Importantly, stress-induced MOMP sometimes spares a minority of mitochondria-containing cells from apoptosis. For instance, sublethal MOMP using overexpression of BH3-mimetic or BH3-only proteins promotes tumorigenesis through caspase-dependent genomic instability (Ichim et al., 2015), countered by regulation of mitochondrial dynamics in HeLa and U2OS cells (Cao et al., 2022). Conversely, blocking caspase activity during MOMP yields potent anti-tumorigenic effects tied to immune responses (Giampazolias et al., 2017). Amplification of *MCL1* and *MYC* has been observed in residual disease of patients with triple-negative breast cancer after they have completed neoadjuvant chemotherapy (Balko et al., 2014). Our own findings underscore the pivotal role of the endogenous MCL1 inhibitor NOXA (PMAIP1) in the induction of cell death upon treatment with antimitotic drugs (Lohard et al., 2020). Briefly, we demonstrated that paclitaxel treatment triggers a proapoptotic secretome, dependent on the cGAS/STING pathway, in multiple breast cancer cell lines and patient-derived breast cancer cells. This secretome then promotes NOXA-dependent apoptosis in cancer cells that are not directly affected by the drugs. Recent studies also reveal antimitotic therapies activating the cGAS/STING pathway directly in breast cancer cells (Zierhut et al., 2019), supporting better response to genotoxic treatments and immunotherapy (Schadt et al., 2019).

Beyond affecting the primary tumor and established metastases, systemic chemotherapy inevitably also affects CTCs. We have previously discussed how cluster formation can help CTCs bypass numerous stressors, increasing their potential to form metastases. Recent research has shed light on the intriguing connection between paclitaxel treatment and the promotion of CTC cluster formation, contributing to paclitaxel treatment evasion (Dashzeveg et al., 2023). Furthermore, it is commonly assumed that DTCs resist chemotherapy because the vast majority are quiescent (Braun et al., 2000). Recently, this notion was challenged by Carlson et al. (2019). Using 4T07 breast cancer cells implanted into syngeneic mice, they demonstrated that the perivascular niche described above protects breast DTCs from chemotherapy-induced cell death independently of the cell cycle. The authors revealed that inhibiting integrin-mediated interactions between DTCs and the perivascular niche sensitizes DTCs to chemotherapy (Carlson et al., 2019).

Significantly, fractional responsiveness to anticancer agents may arise due to tumor heterogeneity. Natural fluctuations in anti- and pro-apoptotic protein levels, as well as the differing activation rates of pro-death or pro-survival responses based on transient cellular states and reversible resistance, contribute to this partial drug responsiveness (Flusberg et al., 2013). Sublethal treatments may spawn drug-tolerant cancer cells, boosting genetic instability and/or metastatic potential, relying, in particular, on the integrated stress response (Kalkavan et al., 2022). These results suggest that systemic chemotherapy treatments in patients with breast cancer could select resistant cancer cells and activate their integrated stress response, resulting in an increase in their capacity to survive the different steps of the metastatic cascade, therefore promoting patient relapse.

### Targeted and immune checkpoint inhibitor therapies

Integrated with conventional chemotherapy and radiotherapy, patients with breast cancer may receive targeted therapies (including immune checkpoint inhibitors) tailored to the tumor characteristics (see Box 1). This comprehensive approach enhances treatment precision and efficacy by inducing cell death pathways specifically in cancer cells, contributing to improved overall patient survival. An example is the use of olaparib in patients with BRCA-mutated triple-negative breast cancer. About 20% of patients with triple-negative breast cancer present mutation in the *BRCA1* or *BRCA2* genes (Wooster and Weber, 2003), which are essential to the repair of double-strand DNA breaks by homologous recombination repair. Poly(ADP-ribose) polymerase (PARP) is an enzyme involved in the repair of DNA single-strand breaks. PARP inhibitors, such as olaparib, were shown to cause synthetic lethality in BRCA-deficient cancer cells due to defective DNA damage repair, resulting in chromosomal instability, cell cycle arrest and subsequent apoptosis (Bryant et al., 2005; Farmer et al., 2005). However, cancer cells can develop resistance to this DNA damage stress, notably by acquiring secondary ‘revertant’ mutations in *BRCA1* or *BRCA2* that restore the open reading frame of the genes (Barber et al., 2013; Edwards et al., 2008). Recently, olaparib was demonstrated to induce osteoclast differentiation, causing bone loss and establishing an immunosuppressive environment, thereby promoting bone metastasis in BRCA wild-type breast cancer cells (Zuo et al., 2020). This suggests that following resistance acquisition to olaparib, DTCs may gain an advantage in forming bone metastases owing to the lingering effects of olaparib on the bone microenvironment.

Another example is the use of tamoxifen in patients with luminal A and B hormone receptor-positive breast cancer. Tamoxifen is a selective estrogen receptor modulator that competes with estrogen for binding to the estrogen receptor as it has a greater affinity to the receptor than its ligand (Shiau et al., 1998). In addition to its cytostatic effect, tamoxifen was shown to induce apoptosis in breast cancer cells by downregulation of BCL2 at both the mRNA and protein levels (Zhang et al., 1999). Although many patients benefit from tamoxifen, resistance is an important clinical problem. Multiple complex mechanisms of *de novo* or acquired resistance to tamoxifen have been described, including the loss of estrogen receptor expression, upregulation of growth factor signaling and estrogen-independent proliferation (reviewed by Rani et al., 2019). Notably, the CD4–Rb–E2F axis was shown to be activated by the estrogen receptor, independently of estrogen binding, thereby promoting estrogen-independent growth (Miller et al., 2011). To inhibit cancer cell growth, CDK4/6 inhibitor (e.g. palbociclib, ribociclib or abemaciclib) treatment combined with endocrine therapy is now the gold-standard therapy for metastatic disease in patients with luminal breast cancer (McAndrew and Finn, 2022).

Patients with HER2-enriched breast cancer benefit from anti-HER2 antibodies including trastuzumab (Slamon et al., 2001). Trastuzumab negatively interferes with signaling downstream of HER2 (Mohan et al., 2016). Moreover, anti-HER2 antibodies were shown to induce cancer cell death through NK recognition and Fc-receptor-mediated antibody-dependent cell-mediated cytotoxicity (Petricevic et al., 2013). Anti-HER2 antibodies therefore target tumor cell biology and the immune response. One resistance mechanism to anti-HER2 therapy involves CDK4/6 activation, suggesting a potential dual-inhibition strategy using trastuzumab and a CDK4/6 inhibitor (Swain et al., 2023). Another major drawback of trastuzumab is its activation of mTORC1, inhibiting autophagy. This may contribute to the secondary cardiotoxic effects of trastuzumab, as impaired autophagy leads to the accumulation of damaged mitochondria and free radicals in cardiomyocytes (Mohan et al., 2016). In contrast, fully active autophagy was shown to promote HER2-driven tumorigenesis (Vega-Rubín-de-Celis et al., 2018). In particular, autophagy was shown to optimize HER2 expression at the tumor cell surface at the expense of HER2 trafficking from the Golgi to endosomes, multivesicular bodies and small extracellular vesicles (Hao et al., 2021). However, this work implies that cell to cell variations in autophagic flux promotes fractional responses to HER2 targeting, which contribute to tumor cell intrinsic adaptation. Of note, heterogeneity in the expression of *HER2* (at the mRNA level) was reported even in cancer cell lines, due to dynamic and non-genetic mechanisms (Gambardella et al., 2022). Interconversions between HER2-negative and -positive CTCs from a patient with ER^+^/HER2 breast cancer were reported, indicating that these dynamic changes may be functional in the blood (Jordan et al., 2016). HER2-targeted antibody drug conjugates may override resistance owing to HER2 expression heterogeneity, provided that they have bystander effects. Additional HER2 inhibitors encompass small-molecule tyrosine kinase inhibitors such as neratinib [inhibiting EGFR, HER2 and HER4 (ERBB4) irreversibly] and tucatinib (HER2 specific), with the ability to penetrate the blood–brain barrier (reviewed by Swain et al., 2023). In patients with HER2-enriched breast cancer with brain metastasis, the upregulation of xCT (SLC7A11), a cystine/glutamate antiporter facilitating cystine import and glutamate export, was observed (Parida et al., 2022). This transporter plays a role in cellular redox homeostasis. The study by Parida et al. (2022) implies that brain metastases in patients with HER2-enriched breast cancer may exhibit resistance to HER2 inhibitors by bolstering reactive oxygen species neutralization through xCT activation.

Patients with triple-negative breast cancer without *BRCA1* or *BRCA2* mutations can benefit from immune checkpoint inhibitors. Unlike melanoma and lung cancer, monotherapy with immune checkpoint inhibitors has shown limited benefit in breast cancer. Although the combination of anti-PD1 or anti-PDL1 with chemotherapies in neoadjuvant or adjuvant settings has shown a benefit in progression-free survival (Cortes et al., 2020; Schmid et al., 2018), not all patients respond to immune checkpoint inhibitors and patients eventually relapse. Identifying biomarkers and enhancing anti-tumor immunity are critical for predicting patient response and improving overall survival. Bassez et al. (2021) showed that the proportions of immunoregulatory dendritic cells expressing PD-L1, macrophages with specific phenotypes (CCR2^+^ or MMP9^+^) and cancer cells expressing major histocompatibility complex class I/II before treatment correlated positively with T cell expansion upon immune checkpoint blockade (Bassez et al., 2021).

More recently, antibody drug conjugates allowing the delivery of highly toxic compounds directly into the tumor have led to the approval of sacituzumab govitecan for triple-negative breast cancer (Bardia et al., 2021) and estrogen-positive disease (Rugo et al., 2022). It consists of a humanized IgG1κ monoclonal antibody targeting TROP2 (TACSTD2), a glycoprotein overexpressed in 80% of poor prognosis breast cancers, coupled to the topoisomerase I inhibitor SN-38 through a hydrolyzable linker. Despite all these therapies, breast cancer metastasis remains uncurable. In stark contrast to localized disease, which can be cured, this underscores the urgent need for therapies targeting metastatic disease. The challenge lies in addressing highly adaptive cancer cells capable of surviving and adapting to multiple stresses.

## Conclusions

Breast carcinoma cells encounter a multitude of challenges as they progress towards forming metastases. These challenges encompass intricate interactions with neighboring cells and immune responses. Moreover, these cells are subjected to an array of treatments designed to eradicate them, including chemotherapy, targeted therapies, surgical interventions and radiotherapies. An important survival mechanism developed by cancer cells to face these challenges is cell clustering. Although our understanding of the impact of cell clustering on intrinsic stresses is growing, the influence of chemotherapy, targeted therapy or immune checkpoint inhibitors on single cells versus clusters in both patients and preclinical models remains unknown and need to be explored.

An alternative strategy for preventing metastasis involves the development of therapies specifically targeting cancer cell clusters. Currently, this approach for breast cancer treatment is being tested in only one ongoing clinical trial (NCT03928210). The objective is to use digitoxin to induce CTC cluster dissociation into single cells, a strategy demonstrated to curtail metastasis in preclinical models (Gkountela et al., 2019). However, the impact of such therapy on DTCs remains to be determined. Furthermore, the possibility that single cancer cells may, under certain conditions, initiate metastasis cannot be ruled out. Single CTCs represent the predominant CTC subtype in patients, underscoring the importance of identifying the molecular prerequisites for single-cell metastasis formation, as it carries a heightened risk for patients.

Another significant challenge lies in targeting dormant DTCs, as most adjuvant therapies target actively dividing cancer cells. A crucial research objective for the future is to develop therapies against this dormant population or for maintaining their quiescence to prevent metastatic resurgence. Investigating the mechanisms that support the viability of latent metastatic cells holds promise for designing novel approaches to target DTCs and inhibit metastasis. Concurrently, advancement of higher-resolution imaging techniques will be indispensable for detecting small lesions or DTCs and monitoring their progression.

Metastases in multiple organs are a common presentation among patients. Although distinct molecular mechanisms have been identified in different metastatic sites, the question of whether treatment should be tailored to the metastatic organ remains unresolved. Developing and optimizing systemic treatments capable of eliminating all metastases, such as immunotherapies, would be highly advantageous for patients. However, variations in microenvironments and metastatic niches across organs influence cancer cell behavior, stromal infiltration and, consequently, their response to treatment. Future research should prioritize investigating the responses of metastases to treatment in various organs.

Lastly, an approach that has gained prominence in recent years involves inducing more immunogenic cell death. Dying cells, including those being eliminated by chemotherapy, release a plethora of antigens that orchestrate innate and adaptive immune responses (Yatim et al., 2015). Although various anticancer drugs exhibit diverse immune effects, their impact on tumor immunogenicity and interactions between the tumor and the immune response hold significant implications for future clinical strategies. Thoughtful combinations of chemotherapy and immunotherapy must be designed to maximize synergy between these approaches, as exemplified by the combination of anti-PD-1 immune checkpoint inhibitors with chemotherapy in a neoadjuvant setting, which has demonstrated enhanced treatment outcomes in breast cancer (Bardia et al., 2021; Shahbandi et al., 2022). Taken together, the research we discussed in this Review shows how complex the process of metastasis is, and although this complexity represents a significant challenge to the field, it also presents opportunities for meaningful clinical progress.
